# Intrusion errors moderate the relationship between blood glucose and regional cerebral blood flow in cognitively unimpaired older adults

**DOI:** 10.1007/s11682-021-00495-8

**Published:** 2021-08-20

**Authors:** Kelsey R. Thomas, Alexandra J. Weigand, Isabel H. Cota, Emily C. Edmonds, Christina E. Wierenga, Mark W. Bondi, Katherine J. Bangen, for the Alzheimer’s Disease Neuroimaging Initiative

**Affiliations:** 1 Research Service, VA San Diego Healthcare System, 3350 La Jolla Village Drive (151), San Diego, CA USA; 2 Department of Psychiatry, University of California, San Diego, La Jolla, CA USA; 3 San Diego State University/University of California, San Diego Joint Doctoral Program in Clinical Psychology, San Diego, CA USA; 4 Psychology Service, VA San Diego Healthcare System, San Diego, CA USA

**Keywords:** Cerebral blood flow, Glucose, Neuropsychology, Process scores, Intrusion errors, Alzheimer’s disease

## Abstract

Regional cerebral blood flow (CBF) has a complex relationship with cognitive functioning such that cognitively unimpaired individuals at risk for Alzheimer’s disease (AD) may show regional hyperperfusion, while those with cognitive impairment typically show hypoperfusion. Diabetes and word-list intrusion errors are both linked to greater risk of cognitive decline and dementia. Our study examined associations between fasting blood glucose, word-list intrusion errors, and regional CBF. 113 cognitively unimpaired older adults had arterial spin labeling MRI to measure CBF in a priori AD vulnerable regions: medial temporal lobe (MTL), inferior parietal lobe (IPL), precuneus, medial orbitofrontal cortex (mOFC), and pericalcarine (control region). Hierarchical linear regressions, adjusting for demographics, vascular risk, and reference CBF region, examined the main effect of blood glucose on regional CBF as well as whether intrusions moderated this relationship. Higher glucose was associated with higher CBF in the precuneus (β = .134, 95% CI = .007 to .261, *p* = .039), IPL (β = .173, 95% CI = .072 to .276, *p* = .001), and mOFC (β = .182, 95% CI = .047 to .320, *p* = .009). There was no main effect of intrusions on CBF across regions. However, the glucose x intrusions interaction was significant such that having higher glucose levels and more intrusion errors was associated with reduced CBF in the MTL (β = -.186, 95% CI = -.334 to -.040, *p* = .013) and precuneus (β = -.146, 95% CI = -.273 to -.022, *p *= .022). These findings may reflect early neurovascular dysregulation, whereby higher CBF is needed to maintain unimpaired cognition in individuals with higher glucose levels. However, lower regional CBF in unimpaired participants with both higher glucose *and* more intrusions suggests a failure in this early compensatory mechanism that may signal a decrease in neural activity in AD vulnerable regions.

**Supplementary Information:**

The online version contains supplementary material available at 10.1007/s11682-021-00495-8.

## Introduction

The co-occurrence of cerebrovascular and Alzheimer’s disease (AD)-related pathologies are extremely common (Kapasi et al., 2017). Growing evidence suggests that cerebrovascular changes may either accelerate AD-related cognitive decline or be a defining feature of AD (Bangen et al., 2015, 2017; Lee et al., 2016; Zlokovic, 2011). One indicator of cerebrovascular function is cerebral blood flow (CBF), which has consistently shown a complex relationship with cognitive functioning across the AD continuum such that cognitively unimpaired (CU) individuals at risk for AD may show regional hyperperfusion, while those with frank cognitive impairment show hypoperfusion (Bangen et al., 2012; Fleisher et al., 2009; Mattsson et al., 2014; Thomas et al., 2021; Wierenga et al., 2014).

Type 2 diabetes mellitus (T2DM) is a vascular/metabolic disease that is a risk factor for mild cognitive impairment (MCI) and dementia, including AD dementia (Livingston et al., 2020; Luchsinger et al., 2007). Prior work has shown that T2DM is associated with reduced CBF (Bangen et al., 2018) and also modifies the relationship between AD risk factors (e.g., apolipoprotein E [APOE] ɛ4 allele, subtle cognitive decline, tau), and future functional decline (Thomas, et al., 2020a, b). Given that even pre-diabetes is associated with higher risk of cognitive impairment (Yaffe et al., 2004), the current study examined the relationship between fasting blood glucose (FBG), which is higher among individuals with pre-diabetes and diabetes, and regional CBF.

Several studies have shown that subtle cognitive inefficiencies, measured using neuropsychological process and error scores, can be captured in the preclinical phase of AD (Abulafia et al., 2018; Loewenstein et al., 2018) and that these sensitive scores add prognostic value to predict future progression to MCI/dementia, above and beyond traditional AD biomarkers (Thomas et al., 2018). Intrusion errors, defined as non-target words said during a word-list memory test, have been shown to be particularly sensitive to early AD-related changes (Bondi et al., 1999; Thomas et al., 2018). Intrusion errors are not considered in calculating the total learning, recall, and recognition scores on traditional word-list measures, and are often overlooked in cohort studies of aging and AD. Recent work has demonstrated that participants with subtle cognitive difficulties, including those with impaired process scores such as intrusion errors, had higher hippocampal CBF than CU or MCI participants (Thomas et al., 2021), such that an inverted-U pattern of regional CBF changes was observed across diagnostic stages.

Regional CBF appears to be an early biomarker of risk for AD-related declines and has been previously associated with both T2DM and subtle cognitive difficulties. In turn, both T2DM and intrusion errors have been shown to confer greater risk of cognitive decline and progression to MCI/dementia. Thus, the current study aimed to examine the associations between FBG, intrusion errors, and CBF in regions vulnerable to early AD pathology. Furthermore, the moderating effect of intrusion errors on the associations between FBG and regional CBF was examined. We hypothesized that having both higher FBG and more intrusion errors would be associated with lower regional CBF due to a failure in expected neural compensation.

## Methods

Data used in the preparation of this article were obtained from the Alzheimer’s Disease Neuroimaging Initiative (ADNI) database (adni.loni.usc.edu). The ADNI was launched in 2003 as a public–private partnership, led by Principal Investigator Michael W. Weiner, MD. The primary goal of ADNI has been to test whether serial magnetic resonance imaging (MRI), positron emission tomography (PET), other biological markers, and clinical and neuropsychological assessment can be combined to measure the progression of mild cognitive impairment (MCI) and early Alzheimer’s disease (AD). For up-to-date information, see www.adni-info.org.

### Participants

Detailed inclusion criteria for ADNI have been reported elsewhere (Petersen et al., 2010). Briefly, at enrollment, participants were aged 55–90 years, had > 5 years of education or work-history equivalent, had a Geriatric Depression Scale < 6, were fluent in English or Spanish, and were generally healthy (Hachinski score < 5). For this study, all participants denied a clinical history of stroke and were considered cognitively unimpaired (CU), which was defined as not having dementia based on the baseline ADNI assessment (McKhann et al., 1984; Petersen et al., 2010) as well as not being classified as having MCI based on Jak/Bondi actuarial neuropsychological criteria (Bondi et al., 2014; Jak et al., 2009; see *Supplemental Methods* for description of criteria).

The current study included 113 CU participants from ADNI GO/ADNI 2 cohorts who had arterial spin labeling (ASL) MRI data within 12 months of their baseline visit, [^18^F]-fluorodeoxyglucose (FDG)-PET imaging, and FBG data available. Sample characteristics are shown in Table 1.

**Table 1:** Sample (*N* = 113) demographic and descriptive characteristics

	Mean or %	SD or N	Range
Age, years	70.75	6.39	59.50–84.70
Education, years	16.58	2.48	12–20
Female	53.1%	*N* = 60	-
White	94.7%	*N* = 107	-
Hispanic/Latino	4.4%	*N* = 5	-
APOE ε4 carrier	33.6%	*N* = 38	-
MMSE	29.04	1.08	25–30
FDG-PET SUVR	1.30	0.12	0.98–1.66
CSF p-tau/Aβ positive*	26.8%	*N* = 26	-
BMI	27.00	4.57	18.71–51.35
Systolic blood pressure	135.22	16.72	101–195
Diastolic blood pressure	74.54	9.84	50–102
Pulse pressure	60.68	15.18	34–109
FBG, mg/dL	98.90	16.71	55–154
Diabetes	12.4%	*N* = 14	-
On hypoglycemic medication	5.3%	*N* = 6	-
Prediabetes (FBG > 100 mg/dL)	33.6%	*N* = 38	-
Total intrusion errors	4.00	4.40	0–24
CBF by ROI (mL/100 g/min)			
MTL	26.54	7.00	8.11–46.51
Precuneus	31.29	10.27	8.49–57.46
Inferior Parietal Lobe	31.84	9.83	12.14–67.77
Medial Orbital Frontal Cortex	23.92	7.34	7.40–47.71
Pericalcarine	42.93	11.96	16.49–76.70
MTL volume (mm^3^)	5645.14	705.03	3766.00–7027.00
Cortical Thickness (mm)			
Precuneus	2.31	0.12	1.91–2.65
Inferior Parietal Lobe	2.41	0.14	1.96–2.68
Medial Orbital Frontal Cortex	2.32	0.12	2.04–2.71
Pericalcarine	1.55	0.11	1.34–1.93

*APOE* = apolipoprotein E; *MMSE* = Mini Mental State Exam; *FDG*-*PET* = [18F] fluorodeoxyglucose positron emission tomography; *CSF* = cerebrospinal fluid; p-tau/Aβ = phosphorylated tau/β-amyloid ratio; *BMI* = body mass index; *FBG* = fasting blood glucose; *MTL* = medial temporal lobe. All participants on hypoglycemic medications were taking Metformin; there were no participants on insulin

^*^available on subsample only (*n* = 97)

### Structural and arterial spin labeling MRI

Detailed information describing MRI data acquisition, processing, and analysis is available online at http://adni.loni.usc.edu/. Briefly, the T1-weighted 3D MPRAGE structural MRI and ASL scan were done at the same visit and performed on a 3.0 Tesla scanner (Siemens).

Pulsed ASL scans were acquired using QUIPS II with thin-slice T11 periodic saturation sequence (“Q2TIPS”) with echo-planar imaging (Luh et al., 1999). The sequence incorporated the following set parameters: inversion time of arterial spins (T11) = 700 ms, total transit time of the spins (T12) = 1900 ms, tag thickness = 100 mm, tag to proximal slice gap = 25.4 mm, repetition time = 3400 ms, echo time = 12 ms, field of view = 256 mm, 64 × 64 matrix, 24 4-mm thick axial slices [52 tag + control image pairs], time lag between slices 22.5 ms. Additional ASL data collection and processing methods used to derive CBF maps are in *Supplemental Methods*.

FreeSurfer (version 5.1; http://surfer.nmr.mgh.harvard.edu/fswiki) was used to skull-strip, segment, and parcellate the structural scans. FreeSurfer-derived anatomical regions of interest (ROIs) were applied and used to extract mean CBF and volume/cortical thickness for each participant. Mean CBF corrected for partial volume effects was extracted for each ROI and reference region for each hemisphere separately and any hemispheric differences in the main outcomes are reported in the results. To reduce the number of comparisons, however, averaged bilateral estimates for each ROI were used as the outcome variable in our models. We examined the following five a priori ROIs: (1) medial temporal lobe (MTL), which included the hippocampus and entorhinal cortex, (2) precuneus, (3) inferior parietal lobe (IPL), (4) medial orbitofrontal cortex (mOFC), and (5) pericalcarine cortex. The first four ROIs were chosen given prior work showing these regions are vulnerable to early AD-related changes and have associations between CBF and AD severity (Bangen et al., 2017; Dickerson et al., 2011; Mattsson et al., 2014; Thomas et al., 2021; Yew & Nation, 2017). The pericalcarine ROI was selected as a control region to examine specificity of any results given that there are no expected changes in AD (Mattsson et al., 2014; Sanchez et al., 2020). Consistent with other CBF work in ADNI (Mattsson et al., 2014; Thomas et al., 2021; Yew & Nation, 2017), CBF of the precentral gyrus was selected to serve as a covariate in the models, as it is not thought to be impacted in early AD, allowing for adjustment of expected individual differences in CBF that are likely not due to AD pathologies. If participants were missing baseline ASL data but had ASL data within the first year of their baseline visit, the first occasion of ASL data was used in our analyses.

### Cerebral metabolism

FDG scanning began 30–60 min after intravenous injection of an approximately 5 mCi dose of tracer. PET images were spatially normalized to a Montreal Neurological Institute (MNI) PET template. In line with prior FDG-PET studies (Jagust et al., 2010; Landau et al., 2011), a composite meta-ROI was comprised of standardized uptake value ratios (SUVRs) from the left and right angular gyri, left and right middle/inferior temporal gyri, and bilateral posterior cingulate gyrus. The meta-ROI was intensity normalized by dividing by the mean for a pons and cerebellum reference region (Landau et al., 2011). The FDG-PET meta-ROI was included as a covariate so associations with CBF could be considered independent from this widely used biomarker of cerebral metabolism and AD risk.

### Intrusion errors

The Rey Auditory Verbal Learning Test (AVLT) is a 15-item word-list learning and memory test that includes 5 learning trials, an interference trial with different words, a short-delay free recall trial, a 30-min delay free recall trial, and a delayed recognition. The *intrusion errors* score is the total number of extra-list intrusion errors produced across all learning and delay free recall trials. Prior work has shown that AVLT intrusion errors predict progression from CU to MCI and dementia, after adjusting for neuropsychological total scores (Thomas et al., 2018). Because intrusion errors were non-normally distributed in this sample, a Blom-transformed intrusion errors variable was used in all analyses (Blom, 1958). However, the raw intrusion errors variable was also examined in analyses and results were qualitatively and statistically similar.

### Fasting blood glucose

FBG values were measured as part of the FDG-PET protocol. Participants had blood drawn following a fast of at least 4 h (water only). For sensitivity analyses, T2DM status was defined based on any of the following: a baseline FBG level of > 125 mg/dL (American Diabetes Association, 2020), self-report of a T2DM diagnosis, or use of glucose-lowering agents (Li et al., 2016; Thomas, et al., 2020a, b). There were no participants with Type 1 diabetes.

### Covariates

Covariates were included to determine whether associations of FBG and intrusion errors with regional CBF persisted beyond general vascular and AD risk. Vascular risk was indexed by pulse pressure (a proxy for arterial stiffness, measured as systolic minus diastolic blood pressure; Nation et al., 2013) and body mass index (BMI). APOE ɛ4 carrier status was determined by the presence of at least one ɛ4 allele. A subset of participants had AD markers from cerebrospinal fluid (CSF) data (*n* = 97). AD CSF markers were processed using Elecsys® immunoassays; biomarker positivity was defined using the previously determined ADNI-optimized phosphorylated tau/β-amyloid (p-tau/Aβ) ratio cut-score of > 0.0251 pg/ml (Hansson et al., 2018).

### Statistical analyses

Hierarchical linear regressions were run for each a priori CBF ROI: MTL, IPL, precuneus, mOFC, and pericalcarine (control region). Block 1 included the main effects of FBG and intrusion errors as well as all relevant covariates: age, sex, education, APOE ε4 status, BMI, pulse pressure, FDG-PET, and reference CBF region (precentral gyrus). Block 2 included the FBG x intrusion errors interaction to determine whether intrusion errors moderated the relationship between FBG and regional CBF. Sensitivity analyses were run to determine if 1) the results changed when 1) the 14 participants who meet criteria for T2DM were excluded and 2) also adjusting for CSF p-tau/Aβ ratio positivity. Next, follow-up linear regressions assessing regional gray matter volume (MTL) or cortical thickness (IPL, precuneus, mOFC, and pericalcarine), instead of CBF, in the same a priori ROIs were conducted to determine whether the pattern of structural findings are consistent with the CBF findings. The models were identical to the hierarchical models described above, but did not adjust for precentral gyrus CBF. Intracranial volume was included as a covariate in the MTL volume analyses. Potential multicollinearity of the independent variables was assessed for all models and all variance inflation factor (VIF) values were less than 2.

## Results

### Effects of blood glucose and intrusion errors on CBF

**Fig. 1:**
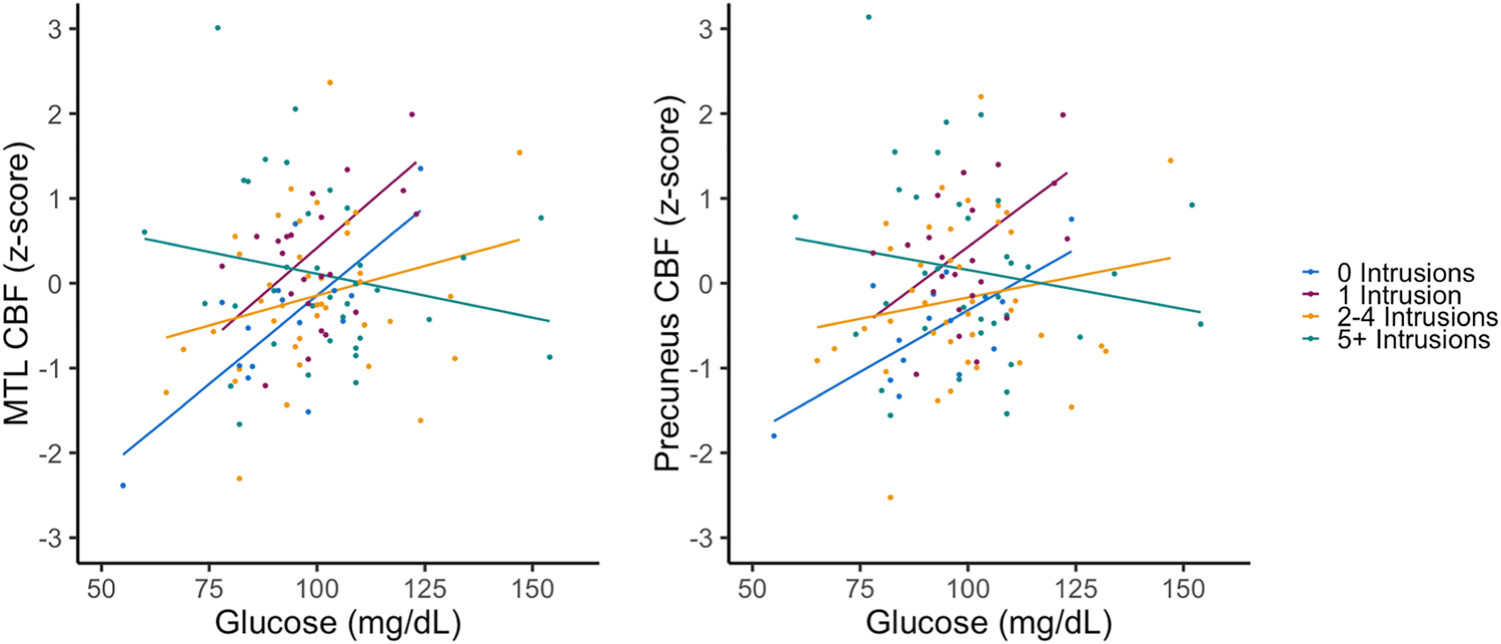
**Intrusions errors moderate the associations between blood glucose and MTL and precuneus CBF.** Intrusion errors were a continuous variable in the models, but intrusions were split into quartiles for visual depiction of the FBG x intrusion errors interaction: 1st quartile = 0 Intrusions; 2nd quartile = 1 intrusion; 3rd quartile = 2–4 intrusions; 4th quartile = 5 + intrusions. Model predicted CBF is shown in z-score metric and includes adjustment for age, sex, education, APOE ε4 carrier status, BMI, pulse pressure, FDG-PET, and reference CBF region (precentral gyrus). MTL = medial temporal lobe; CBF = cerebral blood flow; FBG = fasting blood glucose

Table 2 shows all parameters for the hierarchical linear regressions for each CBF ROI. Briefly, in Block 1, the main effect of FBG was significant such that higher FBG was associated with higher CBF in the precuneus (β = 0.134, 95% CI = 0.007 to 0.261, *p* = 0.039), IPL (β = 0.173, 95% CI = 0.072 to 0.276, *p* = 0.001), and mOFC (β = 0.182, 95% CI = 0.047 to 0.320, *p* = 0.009) regions. There were no significant main effects of intrusion errors on CBF across ROIs. In Block 2, when the FBG x intrusion errors interaction was added to the model, the interaction effect was significant such that intrusion errors moderated the relationship between FBG and MTL and precuneus CBF. Specifically, as shown in Fig. 1, having higher FBG levels and more intrusion errors was associated with reduced CBF in the MTL (β = -0.186, 95% CI = -0.334 to -0.040, *p* = 0.013) and precuneus (β = -0.146, 95% CI = -0.273 to -0.022, *p* = 0.022). The significant results persisted when the false discovery rate was controlled at 0.05. When hemispheric differences were examined, only the FBG x intrusion errors interaction effect differed for the left and right MTL, such that there was a stronger moderating effect of intrusion errors on FBG for the left MTL CBF (β = -0.188, 95% CI = -0.327 to -0.049, *p* = 0.009) relative to the right MTL CBF (β = -0.136, 95% CI = -0.293 to 0.020, *p* = 0.086).

**Table 2:** Effects of hierarchical linear regression models examining associations with a priori CBF regions of interest

	MTL			Precuneus			Inferior Parietal			Medial Orbital Frontal			Pericalcarine (control)		
	β	s.e.	p	β	s.e.	p	β	s.e.	p	β	s.e.	p	β	s.e.	p
Block 1															
Age	.006	.078	.937	-.037	.066	.578	.188	.053	**.001**	.015	.071	.830	-.084	.078	.284
Education	.031	.079	.687	-.042	.067	.525	.091	.054	.087	-.030	.072	.671	.009	.079	.914
Female	.022	.079	.775	.090	.067	.178	.223	.053	**<.001**	-.083	.071	.247	.170	.079	**.035**
APOE ε4 carrier	-.171	.078	**.030**	-.091	.066	.173	-.036	.053	.504	-.093	.071	.193	-.076	.079	.340
BMI	.045	.075	.549	-.046	.064	.474	-.013	.051	.803	-.074	.068	.280	-.003	.075	.963
Pulse pressure	-.026	.079	.739	.040	.067	.555	-.112	.054	**.040**	-.114	.072	.118	.097	.078	.224
FDG-PET	-.007	.079	.929	.093	.067	.163	.108	.054	**.044**	.056	.072	.428	.043	.080	.584
Precentral gyrus CBF	.622	.075	**<.001**	.747	.064	**<.001**	.758	.051	**<.001**	.730	.069	**<.001**	.619	.075	**<.001**
FBG	.131	.076	.084	.134	.064	**.039**	.173	.051	**.001**	.182	.069	**.009**	.081	.076	.285
Intrusion errors	.003	.076	.965	.037	.064	.560	.005	.052	.930	-.073	.069	.288	.044	.075	.565
Block 2															
Age	.011	.076	.887	-.033	.065	.609	.190	.053	**<.001**	.016	.071	.819	-.082	.077	.293
Education	.036	.077	.634	-.038	.066	.554	.093	.053	.078	-.029	.072	.683	.011	.079	.891
Female	-.012	.078	.876	.063	.066	.344	.208	.054	**<.001**	-.091	.073	.213	.146	.080	.071
APOE ε4 carrier	-.165	.076	**.033**	-.086	.065	.189	-.033	.053	.537	-.091	.071	.202	-.073	.079	.356
BMI	.034	.073	.643	-.054	.062	.385	-.018	.051	.730	-.077	.069	.266	-.012	.074	.879
Pulse pressure	-.040	.077	.607	.029	.066	.660	-.118	.054	**.030**	-.117	.072	.110	.087	.078	.268
FDG-PET	.022	.078	.778	.115	.066	.080	.120	.054	**.026**	.063	.073	.383	.062	.080	.429
Precentral gyrus CBF	.604	.074	**<.001**	.732	.063	**<.001**	.750	.051	**<.001**	.726	.069	**<.001**	.608	.075	**<.001**
FBG	.134	.074	.072	.135	.063	**.033**	.174	.051	**.001**	.183	.069	**.009**	.083	.075	.269
Intrusion errors	.007	.074	.929	.040	.063	.526	.006	.051	.908	-.072	.069	.294	.046	.074	.541
FBG x Intrusion errors	-.186	.074	**.013**	-.146	.063	**.022**	-.081	.051	.118	-.043	.069	.537	-.123	.075	.104

Bold p-values are significant *p* < .05. Block 1 includes all covariates with FBG and intrusion errors as the primary independent variables; Block 2 added the FBG x intrusion errors interaction to the model. *CBF* = cerebral blood flow; *MTL* = medial temporal lobe; *APOE* = apolipoprotein E; *BMI* = body mass index; *FDG*-*PET* = [18F] fluorodeoxyglucose positron emission tomography; *FBG* = fasting blood glucose

### CBF sensitivity analyses

Analyses were re-run after excluding participants with T2DM (*n* = 14). There were no changes to the pattern of FBG x intrusion error interaction results (See Supplemental Table 1). The main effects of FBG on IPL and mOFC CBF also remained significant, however, the main effect of FBG on precuneus CBF was attenuated (β = 0.080, 95% CI = -0.071 to 0.266,* p* = 0.253).

Next, the original analyses were run again after adding in the CSF p-tau/Aβ positivity variable (*n* = 97; See Supplemental Table 2). This additional covariate did not change the pattern of results for the FBG x intrusion errors interaction across any of the CBF ROIs, except the effect on the precuneus was slightly attenuated (β = -0.130, 95% CI = -0.272 to 0.008, *p* = 0.064). For MTL, the main effect of FBG was significant (β = 0.179, 95% CI = 0.023 to 0.325, *p* = 0.025) with the addition of the CSF p-tau/Aβ positivity variable such that higher FBG was associated with higher MTL CBF. The pattern of results for any other main effects of FBG or intrusion errors did not change.

### Gray matter secondary analyses

Linear regressions with the same a priori ROIs as the CBF analyses were conducted with regional brain volume/cortical thickness to determine whether the pattern of structural findings is consistent with the CBF findings. In Block 1, there were neither main effects of FBG nor intrusion errors for any of the 5 ROIs. In Block 2, when the FBG x intrusion errors interaction was added to the model, the interaction effect was also not significant across any of the gray matter ROIs (all *p*s > 0.05; See Supplemental Table 3).

## Discussion

The current study found that higher FBG was associated with higher CBF in the precuneus, IPL, and mOFC (and MTL only when adjusting for CSF p-tau/Aβ positivity). While there was no main effect of intrusion errors on regional CBF, intrusion errors moderated the relationship between FBG and regional CBF such that higher FBG and more intrusion errors were associated with reduced CBF in the MTL and precuneus. These interaction effects were largely similar when CSF AD biomarker status was added to the model and persisted when participants with T2DM were excluded.

The main effects of FBG are consistent with prior work that has found higher regional CBF, particularly in the MTL, in participants at risk for progression to AD (e.g., APOE ɛ4 carriers, those with subtle cognitive decline), but who are not yet considered cognitively impaired (Bangen et al., 2012; Fleisher et al., 2009; Thomas et al., 2021). These findings may reflect early neurovascular dysregulation, whereby higher CBF is needed to maintain CU performance in individuals with higher FBG levels. Conversely, prior work has demonstrated that participants with T2DM show reduced CBF relative to participants without T2DM (Bangen et al., 2018). The reason for these discrepant results may be related to the nature of the samples. For example, ADNI was selected to be a very healthy sample given that only individuals with a modified Hachinski index of < 5 were included at baseline. The sample in Bangen et al. (2018) was recruited from the community and was also quite healthy. However, all of those participants with T2DM also had hypertension and the sample included both CU and MCI participants rather than only CU. Thus, higher FBG levels (consistent with the T2DM diagnosis) plus other risk factors such as MCI or hypertension may be related to hypo- rather than hyperperfusion. Within the current study, all participants were CU, less than half of participants (40.7%) had elevated systolic (> 140 mmHg) or diastolic (> 90 mmHg) blood pressure, and there were only 7 participants with both T2DM and high blood pressure.

The current results found that intrusions errors, a known risk factor for progression to MCI/dementia in ADNI (Thomas et al., 2018), moderated the relationship between FBG and regional CBF in the MTL and precuneus (higher FBG level plus greater intrusion errors was associated with lower CBF). This finding suggests that while higher regional CBF is needed to maintain unimpaired performance in people with higher FBG alone, the combination of higher FBG and high intrusion errors may indicate a failure of early compensatory mechanisms that then result in a decrease in neural activity in AD vulnerable regions (Wierenga et al., 2014). This hypoperfusion, in turn, is then consistent with the known reduced CBF in individuals in the MCI and dementia stages of AD (Bangen et al., 2012; Mattsson et al., 2014) as well as consistent with Zlokovic’s two-hit hypothesis of AD pathogenesis (Zlokovic, 2011). Zlokovic proposed that vascular factors such as T2DM (“hit one”) result in blood–brain barrier dysfunction and a reduction in CBF that then initiate a cascade of events leading to amyloid accumulation (“hit two”); amyloid and/or hypoperfusion can both induce hyperphosphorylation of tau, leading to neurofibrillary tangle formation and ultimately dementia.

Importantly, the current pattern of results was fairly similar when p-tau/Aβ positivity was adjusted for in the models, suggesting that the observed relationships are likely not dependent on AD pathology. The pattern of results also persisted after excluding participants with T2DM, indicating that even pre-diabetes levels of FBG may negatively impact CBF, especially in the context of an additional AD risk factor (i.e., intrusion errors). These findings are consistent with evidence that pre-diabetes can accelerate cognitive decline (Marseglia et al., 2019) and be associated with greater Aβ on PET (Luchsinger et al., 2020) and more severe MTL neurofibrillary tangle pathology among those at genetic risk for AD (Bangen et al., 2016). Finally, the absence of any main effect of FBG or interaction of FBG and intrusion errors on regional gray matter volumes suggests that these regional CBF patterns may be altered earlier and precede the ultimate downstream structural changes of gray matter degeneration that likely would not be observed until significant tau accumulation, supporting CBF/neurovascular dysfunction as an early biomarker of risk for decline.

A strength of the study is that we were able to characterize and adjust for vascular and metabolic markers that are likely associated with FBG levels and could impact CBF, which allowed us to better understand the unique relationship between FBG, intrusion errors, and regional CBF. The current study was limited by the cross-sectional design, which precludes conclusions about causation and temporal sequence. Importantly, our study is also limited in its generalizability beyond this mostly white, highly educated, and very healthy ADNI sample. It is likely that results may change in populations with higher rates of vascular diseases. However, it is noteworthy that we were able to detect associations with CBF despite ADNI participants being such a selected sample. Notwithstanding the current limitations, if replicated, these results may have important research and clinical implications. Since FBG is a potentially modifiable risk factor for cognitive decline (Livingston et al., 2020), there is need for increased monitoring and potential for optimization of interventions to ensure FBG is within a healthy range, even in individuals without T2DM. In future studies, ASL MRI may be a useful tool for measuring the underlying mechanism of FBG intervention and understanding why change in FBG may be related to change in cognition.

Taken together, our results suggest that regional CBF may be a useful biomarker of early neurovascular changes related to FBG levels, but that these associations may vary according to other risk factors such as subtle cognitive changes. Intrusions errors may be a useful behavioral measure of very early cognitive inefficiency in episodic memory in older adults who are still performing within the normal range on neuropsychological measures, and seem to be particularly meaningful in individuals with higher FBG levels.

## Supplementary Information

Below is the link to the electronic supplementary material.

Supplementary file1 (DOCX 56 KB)
