# Supplementary material for: Intrusion errors moderate the relationship between blood glucose and regional cerebral blood flow in cognitively unimpaired older adults
Source: Brain Imaging Behav. 2021 Aug 20;16(1):219–27. doi: 10.1007/s11682-021-00495-8 (PMC8825619; doi:10.1007/s11682-021-00495-8)
Supplement: Supplementary file 1 — Supplementary file1 (DOCX 56 KB) [file 11682_2021_495_MOESM1_ESM.docx]

**Supplemental Materials**

**Supplemental Methods**

**Jak/Bondi MCI criteria:**

Jak/Bondi MCI criteria (Bondi et al., 2014; Jak et al., 2009) considered six neuropsychological test scores, including two *memory* measures [Rey Auditory Verbal Learning Test (AVLT) delayed free recall correct responses and AVLT recognition (hits minus false positives)]; two *language* measures [30-item Boston Naming Test (BNT) total correct and Animal Fluency total score], and two *attention/executive functioning* measures [Trail Making Test (TMT) Parts A and B times to completion]. Participants were considered MCI if they performed >1 SD below the age-/education-/sex-adjusted mean on: (1) 2 neuropsychological measures within the same cognitive domain *or* (2) at least 1 measure across each of the 3 measured cognitive domains.

The cognitive test z-scores were derived based on a sample of CU participants in ADNI who did not progress to MCI for the duration of their study participation (i.e., “robust” controls; N=385). Consistent with our prior work in ADNI (Edmonds et al., 2015; Thomas et al., 2019, 2020) within the robust control group, each neuropsychological score was regressed on age, education, and sex. Next, the resulting regression weights were used to compute a predicted score for each participant on each score. The z-scores were then calculated by subtracting the predicted score from each participant’s actual score and then dividing by the test-specific regression model’s standard error of the estimate.

**ASL Data Collection and Processing**

ADNI used scanners from three different companies (GE, Siemens, and Philips) and ASL data was collected on ADNI GO/2 participants who were scanned only on Siemens scanners. The following justification describes why ASL data was only collected for participants scanned on Siemens scanners was “1) We used only vendor product sequences in ADNI GO/2 (i.e. no works in progress sequences were used, because these require a research license for each site), and some of these sequences were not available as product from all MR vendors at the time ADNI GO/ 2 began, and 2) implementation of these sequences was highly variable across vendors. To optimize uniformity of acquisition we limited each of these sequences to a single vendor” (Jack et al., 2015).

ASL MRI processing was largely automated using a MATLAB pipeline and involved motion correction, aligning each ASL frame to the first frame using a rigid body transformation, and least squares fitting using SPM8 (<http://www.fil.ion.ucl.ac.uk/spm/>). Perfusion-weighted images were computed as the difference of the mean-tagged and mean-untagged ASL data and were intensity scaled to account for signal decay during acquisition and to generate intensities in meaningful physiological units. After geometric distortion correction using Insight Toolkit libraries (Yoo et al., 2002), ASL images were aligned to structural T1 images using FSL (<http://www.fmrib.ox.ac.uk/fsl/>). To minimize the effects of the lower perfusion in white matter on CBF estimates, a partial volume correction was performed that assumed that CBF in gray matter is 2.5 times greater than in white matter; SPM8 was used for tissue segmentation. The partial volume corrected perfusion-weighted images were normalized by the reference image (i.e., an estimate of blood water magnetization) to convert the signal into physical units (mL/100 g tissue/min).

**Supplemental Results**

**Figure 1. Consort Diagram**

**Supplemental Table 1. Effects of sensitivity analyses examining associations with *a priori* CBF regions of interest after excluding participants with diabetes (N=14).**

|  |  | MTL | | | | Precuneus | | | | Inferior Parietal | | | | Medial Orbital Frontal | | | | Pericalcarine (control) | | | |
| --- | --- | --- | --- | --- | --- | --- | --- | --- | --- | --- | --- | --- | --- | --- | --- | --- | --- | --- | --- | --- | --- |
|  | β | | s.e. | p | β | | s.e. | p | β | | s.e. | p | β | | s.e. | p | β | | s.e. | p |  |
| Age | -.004 | | .083 | .960 | -.086 | | .066 | .227 | .189 | | .055 | **.002** | .025 | | .073 | .740 | -.095 | | .084 | .270 |  |
| Education | .048 | | .084 | .567 | -.026 | | .068 | .708 | .102 | | .056 | .078 | -.018 | | .074 | .816 | .031 | | .086 | .720 |  |
| Female | -.020 | | .083 | .816 | .080 | | .067 | .253 | .218 | | .055 | **<.001** | -.097 | | .074 | .206 | .156 | | .086 | .071 |  |
| APOE ε4 carrier | -.182 | | .084 | **.031** | -.103 | | .067 | .144 | -.034 | | .056 | .553 | -.074 | | .074 | .327 | -.052 | | .087 | .541 |  |
| BMI | .027 | | .079 | .744 | -.065 | | .063 | .343 | -.010 | | .052 | .854 | -.081 | | .069 | .273 | .007 | | .080 | .934 |  |
| Pulse pressure | -.016 | | .082 | .852 | .054 | | .066 | .451 | -.126 | | .055 | **.033** | -.126 | | .073 | .106 | .100 | | .083 | .246 |  |
| FDG-PET | .040 | | .083 | .640 | .142 | | .066 | .048 | .129 | | .055 | **.027** | .042 | | .073 | .580 | .065 | | .085 | .447 |  |
| Precentral gyrus CBF | .585 | | .083 | **<.001** | .719 | | .066 | **<.001** | .761 | | .055 | **<.001** | .740 | | .073 | **<.001** | .596 | | .084 | **<.001** |  |
| FBG | .075 | | .106 | .368 | .080 | | .085 | .253 | .124 | | .070 | **.031** | .163 | | .093 | **.032** | .027 | | .108 | .752 |  |
| Intrusion errors | .016 | | .079 | .843 | .087 | | .064 | .210 | .005 | | .053 | .923 | -.085 | | .070 | .258 | .052 | | .080 | .530 |  |
| FBG x Intrusion errors | -.184 | | .085 | **.030** | -.149 | | .068 | **.035** | -.081 | | .057 | .156 | -.052 | | .075 | .489 | -.104 | | .086 | .220 |  |

Bold p-values are significant p<.05. Block 1 includes all covariates with FBG and intrusion errors as the primary independent variables; Block 2 added the FBG x intrusion errors interaction to the model. CBF=cerebral blood flow; MTL=medial temporal lobe; APOE=apolipoprotein E; BMI=body mass index; FDG-PET= [18F] fluorodeoxyglucose positron emission tomography; FBG=fasting blood glucose.

**Supplemental Table 2. Effects of sensitivity analyses examining associations with *a priori* CBF regions of interest after adjusting for CSF p-tau/Aβ positivity.**

|  |  | MTL | | | | Precuneus | | | | Inferior Parietal | | | | Medial Orbital Frontal | | | | Pericalcarine (control) | | | |
| --- | --- | --- | --- | --- | --- | --- | --- | --- | --- | --- | --- | --- | --- | --- | --- | --- | --- | --- | --- | --- | --- |
|  | β | | s.e. | p | β | | s.e. | p | β | | s.e. | p | β | | s.e. | p | β | | s.e. | p |  |
| Age | .104 | | .084 | .218 | -.072 | | .079 | .346 | .136 | | .063 | .028 | .009 | | .084 | .911 | -.143 | | .092 | .119 |  |
| Education | .033 | | .083 | .691 | -.026 | | .077 | .729 | .132 | | .062 | .030 | .011 | | .083 | .893 | -.009 | | .093 | .921 |  |
| Female | -.041 | | .083 | .629 | .046 | | .078 | .552 | .221 | | .063 | **<.001** | -.082 | | .084 | .311 | .122 | | .095 | .196 |  |
| APOE ε4 carrier | -.165 | | .087 | .057 | -.128 | | .081 | .102 | -.077 | | .066 | .220 | -.047 | | .087 | .566 | -.184 | | .096 | .051 |  |
| BMI | .063 | | .076 | .416 | -.050 | | .071 | .479 | -.014 | | .057 | .803 | -.078 | | .076 | .286 | -.013 | | .084 | .879 |  |
| Pulse pressure | -.138 | | .079 | .091 | .020 | | .074 | .786 | -.140 | | .059 | **.020** | -.158 | | .079 | **.044** | .071 | | .086 | .417 |  |
| FDG-PET | .020 | | .081 | .811 | .122 | | .076 | .104 | .095 | | .061 | .112 | .052 | | .081 | .506 | .085 | | .091 | .346 |  |
| Precentral gyrus CBF | .601 | | .073 | **<.001** | .739 | | .068 | **<.001** | .749 | | .055 | **<.001** | .746 | | .073 | **<.001** | .616 | | .080 | **<.001** |  |
| CSF p-tau/Aβ positive | -.038 | | .090 | .674 | .048 | | .084 | .564 | .066 | | .068 | .316 | .040 | | .090 | .648 | .138 | | .099 | .160 |  |
| FBG | .179 | | .076 | **.025** | .152 | | .071 | **.036** | .179 | | .057 | **.002** | .160 | | .076 | **.035** | .134 | | .084 | .116 |  |
| Intrusion errors | .012 | | .075 | .879 | .021 | | .070 | .759 | -.008 | | .057 | .879 | -.072 | | .075 | .324 | .031 | | .082 | .705 |  |
| FBG x Intrusion errors | -.162 | | .075 | **.036** | -.130 | | .070 | .064 | -.087 | | .057 | .120 | -.071 | | .075 | .331 | -.090 | | .082 | .277 |  |

Bold p-values are significant p<.05. Block 1 includes all covariates with FBG and intrusion errors as the primary independent variables; Block 2 added the FBG x intrusion errors interaction to the model. CBF=cerebral blood flow; MTL=medial temporal lobe; APOE=apolipoprotein E; BMI=body mass index; FDG-PET= [18F] fluorodeoxyglucose positron emission tomography; CSF=cerebrospinal fluid; FBG=fasting blood glucose.

**Supplemental Table 3. Effects of secondary analyses examining associations with *a priori* gray matter regions of interest**

|  |  | MTL volume | | | | Precuneus cortical thickness | | | | Inferior Parietal cortical thickness | | | | Medial Orbital Frontal cortical thickness | | | | Pericalcarine (control) cortical thickness | | | |
| --- | --- | --- | --- | --- | --- | --- | --- | --- | --- | --- | --- | --- | --- | --- | --- | --- | --- | --- | --- | --- | --- |
|  | β | | s.e. | p | β | | s.e. | p | β | | s.e. | p | β | | s.e. | p | β | | s.e. | p |  |
| Age | -.415 | | .076 | **<.001** | -.311 | | .096 | **.002** | -.310 | | .095 | **.002** | .107 | | .103 | .303 | -.236 | | .101 | **.023** |  |
| Education | -.097 | | .077 | .209 | .088 | | .098 | .362 | .036 | | .098 | .705 | -.125 | | .105 | .230 | -.031 | | .104 | .764 |  |
| Female | -.224 | | .096 | **.021** | .176 | | .098 | .076 | .220 | | .098 | .026 | -.066 | | .106 | .535 | .045 | | .104 | .667 |  |
| APOE ε4 carrier | -.089 | | .077 | .246 | -.046 | | .097 | .636 | -.011 | | .097 | .907 | -.026 | | .104 | .799 | .049 | | .103 | .633 |  |
| BMI | .140 | | .074 | .059 | .113 | | .093 | .228 | .095 | | .093 | .306 | .060 | | .100 | .550 | .001 | | .099 | .990 |  |
| Pulse pressure | .222 | | .079 | **.006** | -.002 | | .098 | .984 | .012 | | .098 | .902 | -.037 | | .106 | .730 | .105 | | .104 | .318 |  |
| FDG-PET | .243 | | .078 | **.002** | -.030 | | .098 | .756 | .024 | | .098 | .806 | .086 | | .106 | .412 | .040 | | .104 | .700 |  |
| Intracranial volume | .321 | | .095 | **.001** | - | | - | - | - | | - | - | - | | - | - | - | | - | - |  |
| FBG | .026 | | .074 | .721 | .148 | | .094 | .116 | .105 | | .094 | .260 | .102 | | .101 | .312 | .057 | | .099 | .570 |  |
| Intrusion errors | -.133 | | .074 | .073 | -.053 | | .093 | .572 | -.094 | | .093 | .313 | -.048 | | .100 | .632 | .084 | | .099 | .396 |  |
| FBG x Intrusion errors | -.003 | | .074 | .967 | -.018 | | .094 | .848 | .087 | | .094 | .349 | .044 | | .101 | .663 | .049 | | .099 | .623 |  |

Bold p-values are significant p<.05. Block 1 includes all covariates with FBG and intrusion errors as the primary independent variables; Block 2 added the FBG x intrusion errors interaction to the model. MTL=medial temporal lobe; APOE=apolipoprotein E; BMI=body mass index; FDG-PET= [18F] fluorodeoxyglucose positron emission tomography; FBG=fasting blood glucose.

**Supplement References**

Bondi, M. W., Edmonds, E. C., Jak, A. J., Clark, L. R., Delano-Wood, L., McDonald, C. R., Nation, D. A., Libon, D. J., Au, R., Galasko, D., Salmon, D. P., & Initiative, for the A. D. N. (2014). Neuropsychological Criteria for Mild Cognitive Impairment Improves Diagnostic Precision, Biomarker Associations, and Progression Rates. *Journal of Alzheimer’s Disease*, *42*(1), 275–289. https://doi.org/10.3233/JAD-140276

Edmonds, E. C., Delano-Wood, L., Clark, L. R., Jak, A. J., Nation, D. A., McDonald, C. R., Libon, D. J., Au, R., Galasko, D., Salmon, D. P., & Bondi, M. W. (2015). Susceptibility of the conventional criteria for mild cognitive impairment to false-positive diagnostic errors. *Alzheimer’s & Dementia*, *11*(4), 415–424. https://doi.org/10.1016/j.jalz.2014.03.005

Jack, C. R., Barnes, J., Bernstein, M. A., Borowski, B. J., Brewer, J., Clegg, S., Dale, A. M., Carmichael, O., Ching, C., DeCarli, C., Desikan, R. S., Fennema-Notestine, C., Fjell, A. M., Fletcher, E., Fox, N. C., Gunter, J., Gutman, B. A., Holland, D., Hua, X., … Weiner, M. (2015). Magnetic resonance imaging in Alzheimer’s Disease Neuroimaging Initiative 2. *Alzheimer’s & Dementia*, *11*(7), 740–756. https://doi.org/10.1016/j.jalz.2015.05.002

Jak, A. J., Bondi, M. W., Delano-Wood, L., Wierenga, C., Corey-Bloom, J., Salmon, D. P., & Delis, D. C. (2009). Quantification of Five Neuropsychological Approaches to Defining Mild Cognitive Impairment. *The American Journal of Geriatric Psychiatry*, *17*(5), 368–375. https://doi.org/10.1097/JGP.0b013e31819431d5

Thomas, K. R., Bangen, K. J., Weigand, A. J., Edmonds, E. C., Wong, C. G., Cooper, S., Delano-Wood, L., & Bondi, M. W. (2020). Objective subtle cognitive difficulties predict future amyloid accumulation and neurodegeneration. *Neurology*, *94*(4), e397–e406. https://doi.org/10.1212/WNL.0000000000008838

Thomas, K. R., Edmonds, E. C., Eppig, J. S., Wong, C. G., Weigand, A. J., Bangen, K. J., Jak, A. J., Delano-Wood, L., Galasko, D. R., Salmon, D. P., Edland, S. D., & Bondi, M. W. (2019). MCI-to-normal reversion using neuropsychological criteria in the Alzheimer’s Disease Neuroimaging Initiative. *Alzheimer’s & Dementia*, *15*(10), 1322–1332. https://doi.org/10.1016/j.jalz.2019.06.4948

Yoo, T. S., Ackerman, M. J., Lorensen, W. E., Schroeder, W., Chalana, V., Aylward, S., Metaxas, D., & Whitaker, R. (2002). Engineering and algorithm design for an image processing Api: A technical report on ITK--the Insight Toolkit. *Studies in Health Technology and Informatics*, *85*, 586–592.
